# Advances in the Biology, Detection Techniques, and Clinical Applications of Circulating Tumor Cells

**DOI:** 10.1155/2022/7149686

**Published:** 2022-09-02

**Authors:** Siwen Wu, Shubi Zhao, Dawei Cui, Jue Xie

**Affiliations:** ^1^Department of Blood Transfusion, the First Affiliated Hospital, Zhejiang University School of Medicine, Hangzhou, China; ^2^Research Laboratory for Biomedical Optics and Molecular Imaging, Shenzhen Institutes of Advanced Technology, Chinese Academy of Sciences, Shenzhen, China

## Abstract

Circulating tumor cells (CTCs) play a crucial role in tumor recurrence and metastasis, and their early detection has shown remarkable benefits in clinical theranostics. However, CTCs are extremely rare, thus detecting them in the blood is very challenging. New CTC detection techniques are continuously being developed, enabling deeper analysis of CTC biology and potential clinical application. This article reviews current CTC detection techniques and their clinical application. CTCs have provided, and will continue to provide, important insights into the process of metastasis, which could lead to development of new therapies for different cancers.

## 1. Introduction

**Figure 1:**
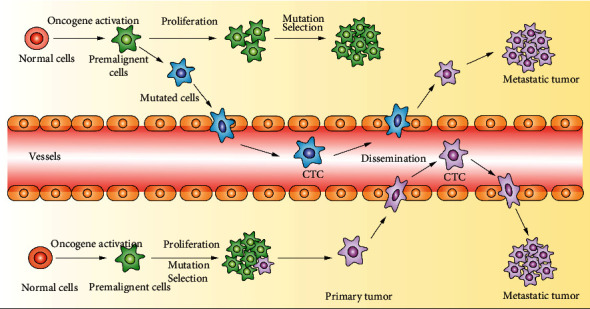
The mechanism of circulating tumor cells (CTCs) driving tumor metastasis. CTCs refer to all kinds of tumor cells in the peripheral blood. Due to their spontaneous or clinic operation, most of the CTCs undergo apoptosis or are swallowed after entering the peripheral blood. A few can escape and are anchored to become metastatic.

Circulating tumor cells (CTCs) were first described by Ashworth in 1869 as a group of tumor cells in the peripheral bloodstream originating from spontaneous solid tumor tissues (primary or metastatic) and a biomarker for cancer diagnosis and progression [1–5]. The mechanism of tumor metastasis caused by circulating tumor cells is shown in Figure 1. Tumor cells shed into the blood *in situ* cause blood-borne metastases [6]. CTCs with an epithelial-mesenchymal transition (EMT) phenotype are invasive enough to pass through the extracellular matrix (ECM), dissociate from the marginal front, and invade the tumor vasculature. CTCs can evade anoikis cell death in circulation. Disseminated tumor cells (DTCs) exhibiting the EMT phenotype undergo intravascular stasis and develop cell protrusions to promote transendothelial migration (TEM) of cancer cells into the metastatic site, where they may stay dormant for some time before colonizing. This allows cancer cells to evade immune surveillance and successfully colonize distant organs. DTCs then acquire the mesenchymal-epithelial transition phenotype to proliferate and form secondary tumors. Cancer cells promote self-growth and colonization of the metastatic site by secreting exosomes that promote their dynamic interaction with the tumor microenvironment [7]. Therefore, CTCs provide cellular evidence for metastasis and are useful biomarkers for cancer progression in most cancer patients [8]. Several biological characteristics contribute to the shedding of CTCs by the primary tumor and their role in metastasis. Generally, EMT promotes the formation and metastasis of CTCs. Metastasis is driven by cytokines, proteins, and transforming growth factor (TGF)-*β*-Smad signaling. TGF-*β* promotes metastasis by reducing the expression of epithelial cadherin (E-cadherin). On the other hand, the resistance of A-kinase anchoring protein 8 (AKAP8) to EMT can inhibit breast cancer metastasis. The infiltration of CTCs into the metastatic site is a complicated process. In addition to producing EMT and proteases, endothelial cells (ECs) secrete CXC chemokine ligand 12 (CXCL12) to promote infiltration and perivascular tumor-associated macrophages (TAMs) to upregulate epidermal growth factor and matrix metalloproteinase-9 [9]. A hardened ECM induces the formation of invasive pseudopodia in cancer cells, enabling them to penetrate the ECM to invade blood vessels. The activity of cancer-associated fibroblasts in the recombinant ECM has been shown to promote drilling and subsequent invasion of tumor cells [7].

CTCs are rare in healthy individuals, and even in patients with malignancies, less than one CTC per 10^5^ to 10^7^ peripheral blood mononuclear cells (PBMCs) could be observed. Thus, isolation and enrichment are often the first steps in CTC detection in laboratories and hospitals [10]. There are two classical approaches to separating CTCs from blood samples: physical separation that exploits unique physical properties of CTCs (such as density and size) and immune adhesion, which depends on the high binding affinity of receptors on CTCs to specific antibodies or aptamers [11]. Compared with immune adhesion, physical isolation is a simpler method as it obviates the need for cell labeling. However, the immune adhesion method achieves higher purity in CTC isolation.

Due to technical limitations, few studies have investigated the precision of CTC detection methods. Keller and Pantel discussed how CTC analysis at single-cell resolution provides unique insights into tumor heterogeneity [12]. Martin et al. reviewed preclinical and clinical data on cancer treatment, CTC mobilization, and other factors that may promote metastasis, establishing that advanced therapeutic strategies could benefit patients with locally advanced cancer [13]. However, a systematic review of the occurrence, development, and outcome of CTCs in metastatic cancer is largely lacking. In this review, we present an overview of the biological characteristics of CTCs, current CTC detection techniques, and principles and methods of CTC isolation. Finally, potential applications of CTCs in the treatment of metastatic cancer are proposed.

## 2. Biological Characteristics of CTCs

### 2.1. Cellular Size

Due to high heterogeneity of tumor cells, the pore size in the CellSearch system is typically slightly larger than leukocytes [14]. Other researchers have successfully separated CTCs using size-based platforms that exploit difference in cell sizes. CTCs in prostate cancer are divided into three categories based on size (diameter): very small nuclear CTCs (<8.54 *μ*m), small nuclear CTCs (8.54–14.99 *μ*m), and large nuclear CTCs (>14.99 *μ*m) [3].

Jiang et al. selectively enlarged the size of tumor cells covered with polystyrene microspheres and the modified cells were clearly distinguishable from white blood cells. The modification method had no significant effect on cell survival and proliferation. Using this method, 15 CTC subtypes were detected in 18 cases of colorectal cancer at a concentration of 4–72 CTCs/mL. Thus, this method has great potential in the early diagnosis and individualized treatment of cancer [15]. Zavridou et al. directly compared two different methods of isolating CTCs from head and neck squamous cell carcinoma: a size-dependent microfluidic system and epithelial cell adhesion molecule (EpCAM)-dependent positive selection. The results showed that, in the same blood sample, the label-freesize-dependent CTC separation system had higher sensitivity than the EpCAM-dependent CTC enrichment system [16].

### 2.2. Cellular Density

Density is the physical property exploited in traditional separation and enrichment methods for CTCs [17]. In Ficoll density gradient centrifugation, CTCs, plasma, and monocytes remain in the upper layer, whereas erythrocytes and polymorphonuclear leukocytes settle in the bottom layer. CTCs may occur in both plasma and separation fluid. Thus, some liquids above the red blood cell layer should be collected for enrichment to prevent the loss of CTCs [5]. Huang et al. developed a new density gradient centrifugation method that uses biodegradable gelatin nanoparticles wrapped on silica beads for isolation, release, and downstream analysis of CTCs from colorectal and breast cancer patients. This method has remarkable CTC capture efficiency (>80%), purity (>85%), high CTC-release efficiency (94%), and viability (92.5%) [18]. Thus, this approach provides new opportunities for personalized cancer diagnosis and treatment and may also be useful in developing drug treatment guidelines for cancer.

### 2.3. Heteromorphy in CTCs

Marrinucci et al. conducted cellular morphological evaluation of circulating components of highly metastatic breast cancer. They found highly polymorphic CTCs in breast cancer patients, including CTCs with high and low nuclear-to-cytoplasmic ratios and early and late apoptotic changes. In addition, compared with tumor cells in other sites, the complete morphologic spectrum of cancer cells in primary and metastatic tumors was also present in peripheral blood circulation [19]. Several studies have found various forms of CTCs in peripheral blood existing either independently or in clusters, with some CTCs even interacting with platelets to form a shell around them [20–23]. Aceto et al. found that CTC clusters in breast cancer-bearing mice were shed as whole oligomeric colonies rather than as simply a group of CTCs aggregating in the bloodstream [24, 25].

### 2.4. Proliferation and Apoptosis of CTCs

For tumor cells in circulation, only a few CTCs with high viability and potent metastatic potential survive and colonize distant organs to develop into metastatic foci. CTCs entering the circulatory system have very short survival times, typically less than 24 h, and vary in their indices of proliferation [26]. Driemel et al. found that the high expression of EpCAM was common in cancer cells in the proliferation stage, while the low expression of EpCAM inhibited the proliferation of CTCs [27]. Studies have reported low levels of expression of proliferating nuclear antigen Ki-67 in CTCs, suggesting that most CTCs may remain in a dormant state without entering the cell division cycle [28, 29]. It has also been found that, several years after primary tumorigenesis, dispersed CTCs and micrometastasis niche can remain dormant for a long time during resection of primary tumors [30]. These results suggest that dormant CTCs can be attached to tissues or cell clusters until their activation or that of a certain factor in the isolation procedures. The specific mechanism may be related to the body's immune response.

### 2.5. The Metastatic Portent of Circulating Tumor Cells

**Figure 2:**
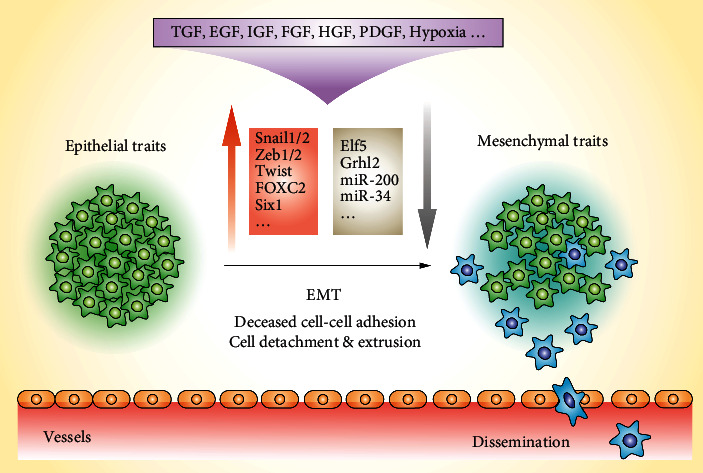
Induction of epithelial-mesenchymal transition (EMT) by various cytokines. It is generally assumed that the metastatic spread of epithelial tumors depends on EMT, a process in which cancer cells lose their polarity and cell-cell adhesion to acquire fibroblast-like features such as migration and invasion.

EMT is a biological process by which epithelial cells acquire a mesenchymal phenotype through a series of biochemical changes [31]. In recent years, accumulating evidence suggests EMT phenomena in the process of cancer cell metastasis [32–34]. In this process, cancer cells lose polarity and their connection with ECM, transforming into fusiform mesenchymal cells, which are easily detached from the tumor cell population. Several ECM-degrading proteases are upregulated in cancer cells with EMT, increasing their invasiveness [32–35]. As shown in Figure 2, the occurrence of EMT in CTCs could result in the loss of specific molecular markers in epithelial cells such as EpCAM and cytokeratin and overexpression of specific molecular markers in interstitial cells such as vimentin and cadherin. These cells have a strong survival advantage and high metastasis and potential for transfer in the blood circulation [17, 36–38]. Based on the EMT stage, CTCs are divided into E-type, M-type, E/M-type, and N (null)-type. Several studies have shown that E/M-type CTCs have enhanced epithelial cell adhesion and extravasation capacity, representing more aggressive subtype of cancer cells with the highest metastatic capacity [39–41]. Additionally, M-type CTCs exhibit enhanced resistance to clinically relevant chemotherapeutics.

## 3. Separation and Enrichment of CTCs

**Figure 3:**
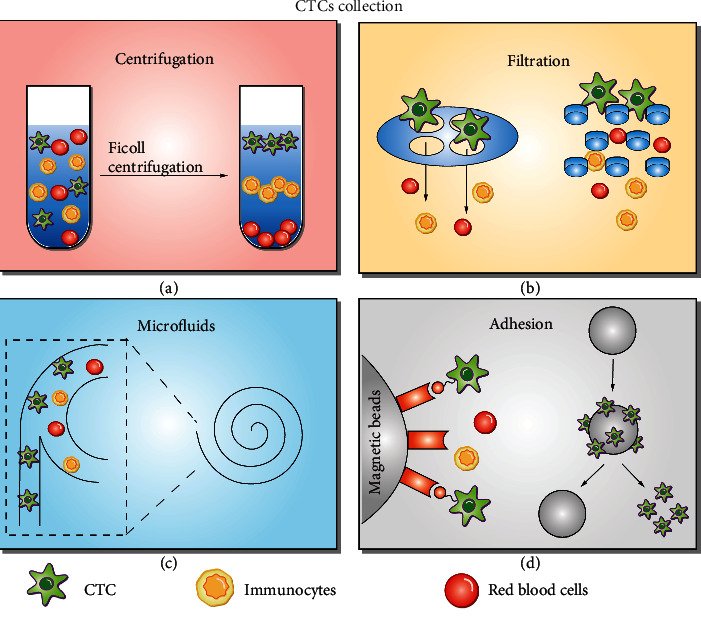
Circulating tumor cell (CTC) enrichment technologies. (a) Density gradient centrifugation. (b) Microfluidic-based separation technology. (c) Different filtration systems depending on the size of blood cells used for separation and enrichment of CTC. (d) Adhesion is dependent on the affinity and specific binding of antibodies or aptamers (e.g., immune adhesion) to the CTC receptor.

There are two major approaches based on the principle of CTC separation and enrichment: physical property separation and affinity-based identification [42, 43]. For the physical property separation method, tumor cells are separated from other cells based on differences in size [44, 45], density [46], deformability, and adhesion between tumor cells and normal blood cells [18, 47]. The affinity-based identification method involves identification of the specific antigen on the surface of cancer cells using antibodies [44, 48], aptamer [49, 50], or E-selectin [51, 52]. Additional details are shown in Figure 3.

### 3.1. Gradient Density Centrifugation

Two centrifugation-based systems are available in the market today: OncoQuick and AccuCyte [43, 53]. After isolation and enrichment with Ficoll-Paque separation fluid, 24 CTCs were detected in fifty-eight 1 mL blood samples from colorectal cancer patients using real-time reverse transcription-polymerase chain reaction (RT-PCR) [53]. Rosenberg et al. reported that using a new OncoQuick system to isolate cancer cells had a 632-fold enrichment effect compared with less than 4-fold enrichment effect using Ficoll-Paque [53]. In addition, 11 CTCs were detected in 37 samples of gastric cancer patients using a combination of OncoQuick and RT-PCR [53]. In another study, 5 and 25 CTCs were detected in 60 cases of early breast cancer patients after immunofluorescence and 63 samples of patients with advanced breast cancer, respectively [2]. Although density-gradient centrifugation is uncomplicated, it lacks specificity and can easily lead to loss of tumor cells without corresponding density.

Therefore, density gradient centrifugation is often used as the first step to separate CTCs and then combined with other methods to specifically bind and separate CTCs. For example, Hu et al. used density gradient centrifugation and magnetic separation based on CD45 antibody to separate CTCs [46]. Different from the traditional negative enrichment, Hu et al. applied the subtraction enrichment and immunostaining fluorescence in situ hybridization (SE-iFISH) strategy to detect CTCs, which effectively removed red blood cells by centrifugation rather than using hypotonic injury [54].

### 3.2. Method for Separation and Capture Based on Cell Size

The method takes advantage of the larger size of CTCs compared with erythrocytes [55]. Isolation by size of epithelial tumor cells (ISET) and ScreenCell systems have been used for clinical trials in melanoma, breast, lung, and pancreatic cancers [56, 57]. For the first time, Zheng et al. used parylene-C to make circular and oval microporous filters, achieving a CTC capture efficiency of 90% [58]. A model for gene analysis and analysis of cells after chip electrolysis developed by Birkhahn et al. [59] was subsequently applied to the detection of exfoliated cells from urinary bladder cancer. Hosokawa et al. integrated nickel microporous sieves made from micro-electroforming into a microfluidic chip. The team also applied the improved nickel microporous sieves to the detection of CTCs in the blood of patients with small-cell lung cancer [60].

To improve the capture efficiency of CTCs, Coumans et al. studied factors affecting the trapping of filter cells [61]. In microfluidic chips, the precise fabrication of shapes and microstructures in microchannels makes it possible to separate and enrich tumor cells on a size-by-size basis [62]. Erythrocytes have stronger deformability and smaller volume, thus can easily cross various microstructures [63]. Niciński et al. proposed a new tool that uses microfluidic devices, photovoltaic (PV)-based SERS activity platform, and shell separation nanoparticles (shins) for simultaneous separation and unlabeled analysis of circulating tumor cells in blood samples. The results demonstrated the potential of SERS-based tools for isolating tumor cells from whole blood samples in a simple and minimally invasive way in a scaled-up detection and molecular identification pipeline [64]. Ohnaga et al. used a microchannel to capture circulating tumor cells in esophageal and breast cancers [65]. Zeinali et al. used a sensitive microfluidic CTC capture device to analyze circulating epithelium and EMT-like CTCs in pancreatic cancer [66].

To capture CTCs larger than the maximum pore size regardless of cell surface expression, blood is filtered through pores (usually 8 *μ*m in diameter). However, the success of this process depends on many factors, including blood flow rate, pore size uniformity, and membrane stiffness. High flow velocity will cause CTC to “squeeze” through pores, causing membrane distortion. A very slow flow rate will lead to excessive accumulation of white blood cells, blood coagulation, and prolonged processing time [67]. Moreover, tumor cells undergoing epithelial-mesenchymal transition (EMT) were smaller than those without EMT characteristics [68]. Therefore, CTCs receiving EMT may not be detected using these technologies. Due to the inherent heterogeneity and dynamic expression of EpCAM and the degradation of cytokeratin during the transformation of epithelial cells into mesenchymal cells, the detection of circulating tumor cells in hepatocellular carcinoma with conventional methods is significantly limited, leading to false-negative detection of such CTCs. Wang et al. reported for the first time the existence of small-sized CTCs (<5 *μ*m WBC) with cytogenetic abnormalities in aneuploid chromosome 8, which is predominantly detected in hepatocellular carcinoma (HCC) patients [69].

### 3.3. Immunomagnetic Beads

Almost all cells in the blood are diamagnetic or weakly magnetic [70]. Therefore, tumor cells are usually labeled with antibody-conjugated magnetic beads or nanoparticles. These antibodies bind primarily to tumor-cellsurface-specific antigens, including some intracellular antigens [71]. The number of CTCs counted using cell search has been used for prognosis of some cancers after metastasis [72–74]. Wu et al. developed a magnetic cell centrifugation platform (MCCP) combining the separation mechanism of magnetically labeled cells with the size-based method and obtained target cells with 97% purity, high throughput of 2 *μ*L/s, and a sample enrichment factor of 66 times [75]. Overall, the performance of the immunomagnetic particle separation method mainly includes the following factors: (1) the expression level and specificity of the target antigen and the binding ability of the corresponding antibody and (2) the efficiency of immunomagnetic particle labeling. Immunomagnetic particles used for cell separation have high recovery and purity and even detect CTCs in one step [76–78].

### 3.4. Chip Technology

**Figure 4:**
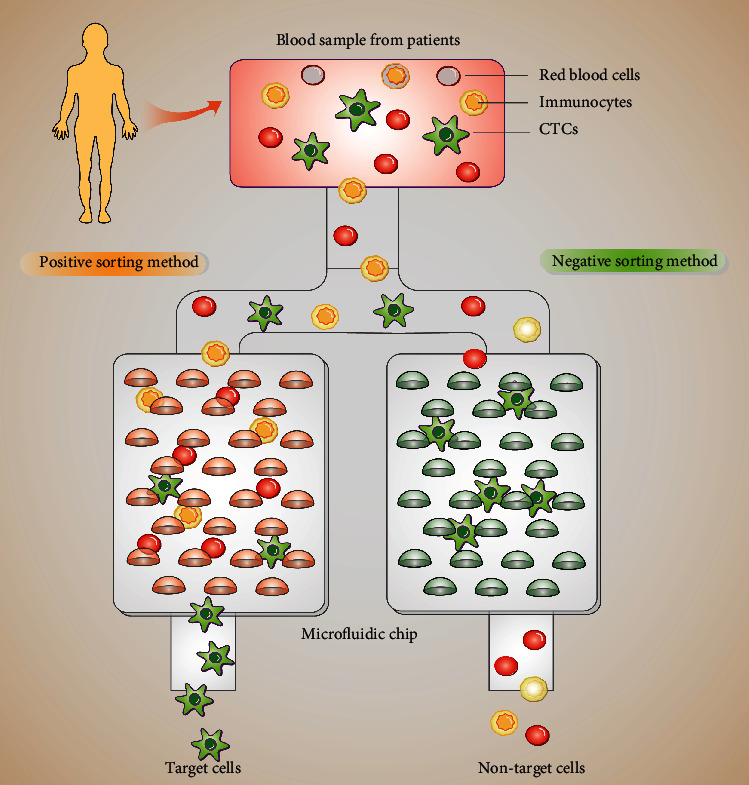
Peripheral blood samples from patients with non-small-cell lung cancer were obtained before any treatment and immediately processed in the circulating tumor cell (CTC) herringbone (HB)-chip that captures anti-EP-CAM-coated column epithelial cell adhesion molecules (left). Negative consumption of untargeted cells by a negative consumption method, including red blood cells and immune cells. Targeted cells such as CTCs were left in the chip for further analysis (right).

In the 1990s, Manz et al. proposed a microfluidic chip technology [79]. Commonly used CTC antibodies include human EpCAM and leukocyte common antigen CD45 [80]. Affinity sorting includes two types of capture methods. The first type is the positive sorting method, which directly targets and specifically captures target cells. The second type is the negative sorting method, which involves the capture nontarget cells, which are then discarded. A schematic diagram of the working principle is shown in Figure 4.

#### 3.4.1. Positive Sorting Methods

The traditional affinity sorting method involves direct binding of the antibody to the microfluidic chip channel [81]. Sequist et al. developed the second-generation CTC chip called herringbone (HB)-chip [82]. Compared with first-generation CTC chips, the second-generationHB-chip is easy to use and more efficient, providing comprehensive and easy access to data. Hughes et al. integrated halloysite nanotubes into this chip [83] by immobilizing E-selectin and anti-EpCAM on nanotubes. In this design, E-selectin captures rapidly moving CTCs, whereas anti-EpCAM specifically captures CTCs [84, 85], increasing the purity of the captured CTCs. To simplify the experimental procedures, Stott et al. designed a fishbone-based affinity sorting chip for direct analysis of whole blood samples with a sorting speed of up to 1 mL/h. Captured circulating tumor cells could also be used for other assays or cell culture [86]. Sheng et al. optimized the fishbone structure to achieve a CTC capture efficiency and sorting purity higher than 90% and 84%, respectively [87]. These microfluidic chip technologies have shown good CTC capture capability. However, releasing CTCs from microfluidic chips for subsequent analysis is challenging. Therefore, researchers have introduced magnetic materials into microfluidic chips for CTC sorting [88].

#### 3.4.2. Negative Sorting Methods

EpCAM-based affinity separation cannot be applied to CTCs with weakly expressed or nonexpressed EpCAM in the process of tumor cell metastasis, which leads to the significant decrease or even loss of EpCAM expression. For example, Lee designed a chip called “*μ*-MixMACS”, which greatly increased the number of CTCs detected [89]. Sajay et al. designed a two-step negative CTC sorting platform where the recovered cells remain bioactive and can be further analyzed for protein or nucleic acid content [90]. A CTC-negative enrichment scheme, which utilized the RosetteSep™ CTC Enrichment Cocktail Containing Anti-CD56 to collect CTCs in peripheral blood, was used to monitor the occurrence and disease response to treatment at different time points [91].

Unlike traditional negative enrichment, researchers utilize subtraction enrichment (SE), independent of cell size, cluster, or surface anchor protein expression. The immunostained proteins were proved to be free from the restriction of antigen epitopes inside and outside cells and membrane-related tumor biomarkers. With the clinical application of SE-iFISH, in addition to the traditional tumor cell types, there are more and more accidental discoveries of various phenotypes of CTCs [54]. Zhang et al. have shown that aneuploidy CD31^−^ CTC and CD31^+^ CTEC may be used as a pair of biomarkers for circulating cell tumors to predict patients with non-small-cell lung cancer receiving antiangiogenesis combined therapy [40]. Based on the SE-iFISH strategy, Yang et al. demonstrated that patients with early bladder cancer had more triploid CTCs, tetraploid CTCs, and total circulation endothelial cells (CECs). Various CTC/CEC subtypes may have different potential function to guide the diagnosis, prognosis prediction, and treatment decision of bladder cancer [92]. Li et al. found that the presence of circulating tumor-cell-associated white blood cell (CTC-WBC) clusters is an independent prognostic factor for advanced non-small-cell lung cancer [93].

## 4. Detection of CTCs

### 4.1. Immunocytochemistry

Immunochemistry is a modern technology that binds specific monoclonal antibodies with CTCs, followed by conjugation of a chromogenic reagent to visualize CTCs. The most commonly used monoclonal antibody are anti-CK antibodies, such as epithelial-specific markers (CK) [94], interstitial cell surface markers (Snail1, E47, and Twist) [95], E-cadherin antagonist [96], stem cell markers (CD133+, CD44+, and CD24−), aldehyde dehydrogenase 1 (ALDH1) [97–100], special marker Survivin [101, 102], estrogen receptor (ER) [103], and progesterone receptor (PR) [104]. EpCAM and CK may be lost during epithelial-mesenchymal transition (EMT), leading to the failure of EpCAM- and CK-dependent strategies to detect CTCs. Therefore, Li et al. used SET-iFISH technology to enrich and characterize CTCs in advanced gastric cancer (AGC) and obtained a higher positive detection rate than that obtained using EpCAM-dependent detection strategies (CellSearch) [105]. In addition, Li et al. captured CTCs in AGC through SE-iFISH and found that cHER2 phenotype is useful to understanding the treatment resistance of AGC patients [106]. Subsequently, scientists used this method to characterize the markers of CTC, such as EpCAM [41], PD-L1 [107], vimentin [40], and CD44 [108].

### 4.2. RT-PCR

Currently, RT-PCR is considered the gold standard in the detection of some viruses because of its high sensitivity [109, 110]. RT-PCR is also widely used in tumor detection [111, 112]. However, selecting optimal RNA markers can be challenging, limiting its efficacy. An ideal RNA marker should have the following characteristics: all types of tumor cells are expressed in peripheral blood leukocytes, nontumor epithelial cells are not expressed, and no illegitimate transcription events [113]. Using qualitative RT-PCR, Wang et al. found that the expression of androgen receptor variant 7 (AR-V7) in CTCs from patients with prostate cancer was associated with drug resistance. The upregulation of AR-V7 led to the enhancement of cancer cell proliferation, suggesting poor patient prognosis [114]. Wei et al. recruited 78 patients with stage I_A2_–II_A1_ cervical cancer who had undergone radical hysterectomy by laparotomy or laparoscopy and selected 34 uterine fibroid patients and 32 healthy subjects as the positive control group and negative control group, respectively. RT-PCR was used to amplify peripheral blood CK19, CK20, and SCC-Ag from total RNA. The results showed that CTCs were highly expressed in both the open surgery group and the laparoscopic radical mastectomy group, with no significant difference between the two groups [115]. Using Survivin, hTERT and hMAM markers to detect CTC in breast cancer patients, Shen et al. found that these markers improved the sensitivity of parallel tests and the specificity of series tests [116]. The molecular spectrum of four genes, including *CK20*, *CK19*, *CEA*, and *GCC*, identified 87.7% of tumor metastases with a false-positive rate of only 2.2% [117].

## 5. Clinical Applications of CTCs

CTCs are a promising biomarker for early disease diagnosis, treatment response and disease progression evaluation, recurrence monitoring, and therapeutic target identification for drug development [118]. Detection of CTCs has been widely used in the diagnosis of early and metastatic cancers (Table 1).

### 5.1. Early Diagnosis and Staging of Cancer

Traditional imaging methods cannot effectively detect early tumor lesions. CTC detection approaches can detect tumor earlier than imaging or clinical manifestations when the lesion is <1 cm, hence facilitate early diagnosis. Besides its role in early tumor diagnosis, CTC is also correlated with tumor grade and TNM stage. Santos et al. found that CTCs have great potential in the early diagnosis of colorectal cancer since they can be detected in the peripheral blood of patients with early-stage colorectal cancer. Therefore, the CTC test may be applied to the diagnosis of colorectal cancer [123]. Clinical staging of colorectal cancer is often based on anatomical alterations of the intestine; however, it is difficult to accurately identify micrometastasis during the prognosis and treatment of patients [124]. Detection of CTCs in the blood does not necessarily indicate the occurrence of metastasis. However, several studies have shown the value of detection of CTCs in the staging of colorectal cancer in clinical practice [125]. Using an advanced CanPatrol CTC enrichment technique and *in situ* hybridization to sort and classify CTCs in blood samples, 90.18% of hepatocellular carcinoma (HCC) patients were found to be CTC positive, even at the early stage of HCC [126]. CTCs were also detected in 2 of 12 patients with hepatitis B virus (HBV) infection, with both patients developing small HCC tumors in less than five months. Another study by Wang et al. implicated CTCs in tumor staging [127]. Recent studies have shown that CTCs also put into good use in hematologic malignancies. Primary plasma cell leukemia (pPCL) is clinically distinguishable from newly diagnosed multiple myeloma (NDMM) based on the proportion of circulating tumor cells of 20% [128]. Zhang et al. also used a technology based on oncolytic herpes-simplex-virus-1 to detect CTCs in non-Hodgkin's lymphoma [129].

### 5.2. Treatment Evaluation and Recurrence Monitoring

Treatment evaluation and recurrence monitoring of CTCs has been extensively studied. Lin et al. measured the number of peripheral blood CTCs before and after NK cell immunotherapy in stage IV non-small-cell lung cancer (NSCLC) patients, providing a useful reference for monitoring any change in NK cell therapeutic effect [130]. Nagrath et al. detected and monitored CTCs using a CTC chip and found that the CTC count of patients with lung and prostate cancer decreased significantly before and after chemotherapy and endocrine therapy. Although the changes in the CTC count due to treatment are affected by the differences between individual patients, they can still be used as a reference for evaluating the efficacy of tumor treatment [131–133]. In some cases, CTCs are more sensitive than imaging, thus they are included in efficacy evaluations [134]. In recent years, several detection techniques have been developed for CTC genotyping as well as detection of crucial gene mutations, such as ER [135], HER2 [136], and TP53 [137]. Thus, these techniques can help clinicians in treatment evaluation and monitoring tumor recurrence. Zhou et al. used PCR and fluorescence-activatedsingle-cell sorting (FACS) to detect levels of EpCAM mRNA^+^ CTCs and CD4^+^CD25^+^Foxp3^+^ Treg cells in 49 HCC patients before surgery. The data showed that CTC/Treg levels were positively correlated with the risk of postoperative recurrence [138].

Due to differences in tumor type and stage among cancer patients and occult, it is difficult to detect metastatic tumor relapse within five years of primary tumor resection. Cancer that persists despite treatment and cannot be detected by current medical imaging modalities is defined as minimal residual disease (MRD), which is in the occult stage of cancer progression. Liquid biopsy methods based on detection of small amounts of circulating tumor cells (CTCs) or trace amounts of circulating cell-free tumor DNA (ctDNA) are now available for MRD detection in patients with various malignant tumors. Monitoring CTCs and ctDNA during postoperative follow-up assessments can detect disease recurrence months earlier than other medical imaging methods. Further characterization of CTCs and ctDNA could provide insights into the molecular evolution of MRD during tumor progression, which has important implications for treatments that delay or even prevent metastatic recurrence. Therefore, the detection of CTCs has become the main method for the assessment of minimal residual disease (MRD) [139].

## 6. Conclusion

Despite the initial promise of CTCs in clinical application, several challenges must be addressed before CTC analysis gains widespread application in clinical practice. At present, CTC cell count and molecular phenotype analysis are applied in practice. More comprehensive characterization of CTC-based genomes, transcriptomes, and proteomes from high-throughput sequencing projects will further benefit clinical applications but also increase the complexity and difficulty of data analysis.

The survival of CTCs in the peripheral blood is a complex process involving multiple factors and mechanisms. It has been reported that hypoxia, autophagy, and secretion of exosomes may affect the prognosis of CTCs. Whether CTCs can be differentiated based on their phenotypes and karyotypes or not and how to formulate individualized treatment regimens for CTCs to resist apoptosis-induced drug resistance are urgent problems that need to be solved. Given the increasing popularity of molecular diagnosis in clinical practice and continuing decline in the cost of the technology, the detection of CTCs will become a powerful and indispensable tool for the diagnosis of circulating tumor cells (tumor DNA) with its advantages of repeatability, mutation detection at the molecular level, noninvasive diagnosis and broad application potential in targeted therapy, efficacy testing, postoperative prognosis, radiotherapy and chemotherapy strategy guidance, as well as in differential diagnosis.

## Figures and Tables

**Table 1:** Clinical applications of CTCs

Cancer type	Patient number	Detection methods	Marker	Significance	Clinical trial no.	Reference
Breast cancer	549	CellSearch	EpCAM, CK	In breast cancer patients with first-line chemotherapy, CTC counts were associated with mortality.	NCT00382018	Paoletti et al. [119]
Prostate cancer	147 mCRPC	VERSA	EpCAM	A transcriptional profile detectable in CTCs can serve as an independent prognostic marker in mCRPC.	NCT01942837, NCT01942837	Sperger et al. [120]
Pancreatic cancer	209 patients	CellSearch	EpCAM	CTC-positive preoperatively (≥1 CTC/7.5 mL) showed a detrimental outcome despite successful tumor resections.	NCT01919151	Hugenschmidt et al. [121]
Colorectal cancer	153	CellSearch	EpCAM, CK	Baseline CTCs ≥ 3 were detected in 19% of the patients. CTC ≥ 3 at baseline and 4 weeks after therapy showed shorter overall survival.	NCT01442935	Bidard et al. [122]

## Data Availability

The data used to support the findings of this study are included within the article.
